# Dissecting the heterogeneity of “in the wild” stress from multimodal sensor data

**DOI:** 10.1038/s41746-023-00975-9

**Published:** 2023-12-20

**Authors:** Sujay Nagaraj, Sarah Goodday, Thomas Hartvigsen, Adrien Boch, Kopal Garg, Sindhu Gowda, Luca Foschini, Marzyeh Ghassemi, Stephen Friend, Anna Goldenberg

**Affiliations:** 1 Department of Computer Science, University of Toronto, Toronto, ON Canada; 2 Vector Institute, Toronto, ON Canada; 3 The Hospital for Sick Children, Toronto, ON Canada; 4 4YouandMe, Seattle, WA USA; 5 Department of Psychiatry, University of Oxford, Oxford, UK; 6 School of Data Science, University of Virginia, Charlottesville, VA USA; 7 Evidation Health Inc, San Mateo, CA USA; 8 Sage Bionetworks, Seattle, WA USA; 9 Institute for Medical Engineering and Science, MIT, Cambridge, MA USA; 10 Department of Electrical Engineering and Computer Science, MIT, Cambridge, MA USA; 11 Canadian Institute for Advanced Research, Toronto, ON Canada; 12 Department of Laboratory Medicine and Pathobiology, University of Toronto, Toronto, ON Canada

**Keywords:** Health care, Computer science

## Abstract

Stress is associated with numerous chronic health conditions, both mental and physical. However, the heterogeneity of these associations at the individual level is poorly understood. While data generated from individuals in their day-to-day lives “in the wild” may best represent the heterogeneity of stress, gathering these data and separating signals from noise is challenging. In this work, we report findings from a major data collection effort using Digital Health Technologies (DHTs) and frontline healthcare workers. We provide insights into stress “in the wild”, by using robust methods for its identification from multimodal data and quantifying its heterogeneity. Here we analyze data from the Stress and Recovery in Frontline COVID-19 Workers study following 365 frontline healthcare workers for 4–6 months using wearable devices and smartphone app-based measures. Causal discovery is used to learn how the causal structure governing an individual’s self-reported symptoms and physiological features from DHTs differs between non-stress and potential stress states. Our methods uncover robust representations of potential stress states across a population of frontline healthcare workers. These representations reveal high levels of inter- and intra-individual heterogeneity in stress. We leverage multiple stress definitions that span different modalities (from subjective to physiological) to obtain a comprehensive view of stress, as these differing definitions rarely align in time. We show that these different stress definitions can be robustly represented as changes in the underlying causal structure on and off stress for individuals. This study is an important step toward better understanding potential underlying processes generating stress in individuals.

## Introduction

Stress is complex and prone to various interpretations, often focusing on physiological, biological, and psychological responses to stressors within an environment. Many mental and physical health conditions are known to be stress-related. Unifying and characterizing clear notions of stress is essential to detecting and mitigating its detrimental effects^1^. Anecdotally, stress is highly heterogeneous both within and between individuals. To date, however, this observation has yet to be sufficiently characterized. While categorizing subjective experiences like stress is challenging, the emerging field of precision medicine has uncovered such inter- and intra-individual heterogeneity of many other disease processes^2,3^. One challenge in studying stress is capturing its nature “in the wild”. Stress occurs at variable frequencies and severity over the lifecourse of an individual, while individual characteristics likely modify an individual’s response to stress in highly varied ways^1^. With the ubiquity and capabilities of Digital Health Tools (DHTs) such as wearable devices, real-time measurement of stress “in the wild” at the individual-level context may be an achievable target. DHTs are capable of assessing both physiological metrics (such as heart rate, heart rate variability, and respiratory rate) as well as potential signs and consequences of stress (changes in sleep quality, mood, and cognition)^4^. Analysis of data from DHTs may help describe and understand the heterogeneity of stress that could lead to more accurate ways of measuring this complex state and in turn, understand how it contributes to chronic conditions^1^.

A major methodological challenge when working with observational sensor data is the low signal-to-noise ratio. In addition, prior approaches leveraging traditional statistical approaches or machine learning to stress detection lack the ability to model the complexity of stress^5–7^, while assuming that stress exists as a singular, universal phenomenon^8^. We need to leverage methods that can accommodate for this complexity (i.e., stress may be different within and between individuals) and also leverage the rich information from DHTs to help characterize it.

We use the PC causal discovery algorithm (named after authors Peter and Clark)^9^ to explore how the causal structure governing an individual’s observations (self-reported symptoms and physiological features from DHTs) change during various potential stress states. Prior studies have examined the connection between sensor data and mental health via predictive modeling with modest results that improve when combining sensor and survey data^5,10^. In contrast, the focus of this analysis is on understanding the underlying process as explained by changes in the causal structure governing observations: how do stressful periods in an individual’s life differ from non-stressful periods?

## Results

### Cohort description

The Stress and Recovery cohort consisted of frontline healthcare workers working with COVID-19 patients from March to December 2020. The cohort was largely middle-aged, female, and Caucasian. A total of 59% of individuals reported that they were diagnosed with a mental health condition (any sleep, anxiety, or mood disorders) (Supplementary Table 2).

### Data completeness

Oura Ring data were quite complete. On average, there were 76 (±32, SD) days of complete observations per individual, spanning a duration of 92 (±31, SD) days of observations including missing days. Survey measures also showed high levels of completeness, which varied across daily, weekly, biweekly, and monthly measures. Completeness tended to decrease with increasing frequency (Supplementary Table 3).

### Periodicity in sensor data

While the periodicity in DHT data may provide meaningful insight into health states, it also poses challenges in isolating other signals, such as stress, especially when certain periodic signals dominate others. We observed strong periodicity in Oura Ring-derived data across individuals. Many individuals have strong 30-day periods of cycling in their data (Supplementary Fig. 3). Particularly for temperature, heart rate, and breath average (respiratory rate) data, this is a largely sex-dependent effect, suggesting that we are observing menstrual cycles. Large-scale studies have also corroborated that these are strong signals that would be observed in a female population^11^. We applied the same Fourier analysis on our encoded stress labels as well. Though Pierson et al. observed menstrual-related periodicity in self-reported measures, we were unable to observe these in our cohort (Supplementary Fig. 4). These findings highlight why applying change point detection approaches would likely be confounded by the rises and falls of physiological cycles and not stress states (results not shown).

### Heterogeneity of stress labels

**Fig. 1:**
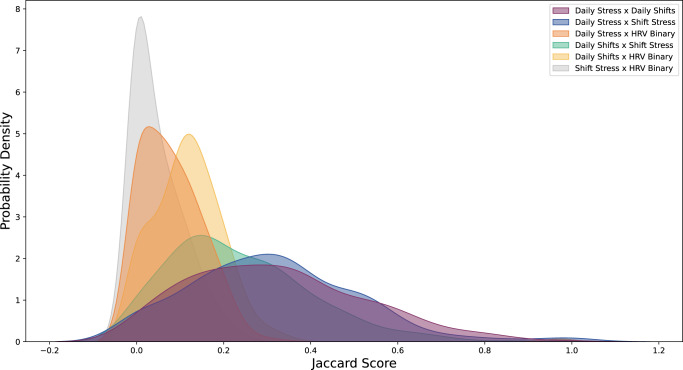
Distributions of pairwise Jaccard similarity scores for each pair of binary stress labels across all individuals. Histograms are normalized to encode a valid probability distribution. Note not all pairs are shown as the measure is symmetric.

Across all pairs of stress labels we used in our analysis, we found poor alignment as represented by low Jaccard scores (Fig. 1), suggesting that potential stress states (according to each label) rarely overlap with each other in time. Interestingly, we found the highest disagreement (lowest Jaccard scores) when comparing HRV Binary to other stress labels. Despite their overall low Jaccard scores, pairs of self-reported stress labels are more likely to overlap (i.e., Daily Stressed and Shift Stress) than two stress labels comparing Oura Ring data and self-reported data (i.e., HRV Binary and Daily Stressed).

### Robustness of stress representations

We learned causal graphs that represent stress and non-stress periods of an individual’s trajectory spanning four different stress labels. From Table 1, we can see that the vast majority of individuals in our cohort had a significant change in the structure of causal graphs during all stress labels compared to a reference distribution of random sampling. Missingness rates, as defined by the overall number of missing values at each time step for each individual, were compared using a two-sample *t*-test to identify the number of individuals who had a significant difference in missingness rates when comparing stress and non-stress time step. Overall, we found few individuals across each stress label (daily stressed: 13, daily shifts: 9, shift stress: 2, HRV binary: 0) who had a significant difference (*p* < 0.05, two-sample *t*-test, one-tailed) in missingness rates, suggesting that missingness rates on and off stress are not driving our results.

**Table 1:** Significant changes in graph similarity measures across the population.

	Graph Similarity Measure		
Stress Label	Connectivity	Intersection/Union	Between Modality Edges
Daily Stressed (*n* = 202)	86.1%	72.8%	74.8%
Daily Shifts (*n* = 294)	86.4%	72.8%	68.4%
Shift Stress (*n* = 72)	91.7%	88.9%	84.7%
HRV Binary (*n* = 292)	84.6%	88.4%	74.3%

Proportion of population showing statistically significant changes in various graph similarity measures on stress compared to a reference distribution of random sampling. Where *n* represents the total number of individuals included in analysis. For graph similarity measure definitions, please see Methods.

The various graph similarity measures we utilized (Methods) show significant changes in the connectivity structure across all stress labels across the population (Table 1). Not only did connectivity structure change (representative of the total number of edges), but the set of edges represented during stress periods was also different as evidenced by changes in Intersection/Union as well as the number of specific edge categories (i.e., between Oura connections) that changed.

### Inter- and intra-individual heterogeneity in stress

**Fig. 2:**
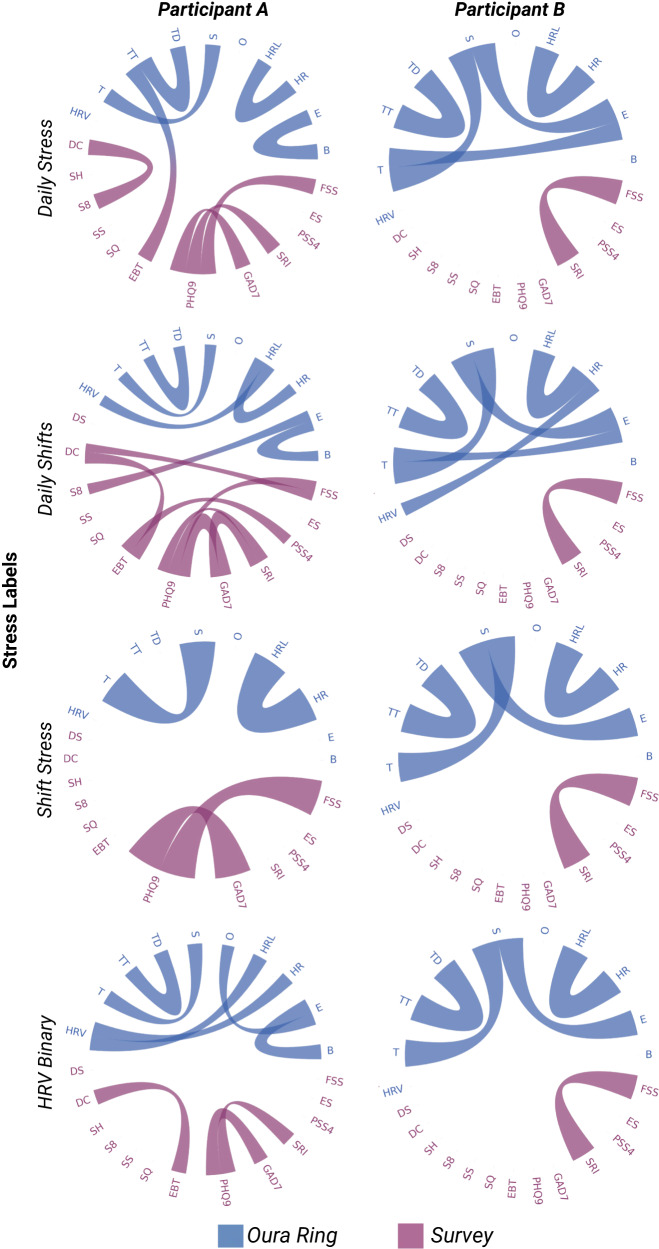
Chord diagrams showing undirected graphs during stress for two different individuals across four different labels. Nodes (features) are on the outer ring, and colored chords represent undirected edges between nodes. Nodes and chords are colored according to the modality they are derived from (blue: Oura Ring, purple: Survey), chords spanning modalities are multicolored. Abbreviations for each feature/node can be found in Supplementary Table 1, along with a complete description of each feature.

We qualitatively analyzed the causal networks during stress across the different stress labels. We found high levels of inter- and intra-individual heterogeneity in causal graph structure on and off stress (Fig. 2). From two random individuals and across four stress labels, causal graphs are highly heterogeneous across different stress labels for the same individual (intra-individual heterogeneity). In addition, comparing two random individuals across the same stress label, we see differences in causal graph structure (inter-individual heterogeneity). Edges also tended to occur within modality types (i.e., Oura Ring to Oura Ring or Survey to Survey), suggesting a high correlation between values from a given type (Table 2).

**Table 2:** Types of edges changing on and off stress.

	Within Modality		Between Modality	
Stress label	Stress	Non-stress	Stress	Non-stress
Daily Stressed	91.5% ± 12.0%	91.1% ± 12.1%	8.0% ± 12.0%	8.9% ± 12.1%
Daily Shifts	92.4% ± 11.2%	92.2% ± 11.1%	7.6% ± 11.2%	7.8% ± 11.1%
Shift Stress	86.1% ± 15.6%	89.5% ± 14.2%	13.9% ± 15.6%	10.5% ± 14.2%
HRV Binary	92.0% ± 12.0%	90.4% ± 13.3%	8.0% ± 12.0%	9.6% ± 13.3%

Percentage of within sensor edges (edges to and from Oura or to and from Survey) and between sensor edges (edges between Oura and Survey) over total number of edges across different stress labels. ± represent standard deviation across individuals.

In order to query possible explanations for these changes in causal structure, we looked at the overall enrichment in time-invariant features (i.e., gender, past mental health history, etc.; Supplementary Table 4) between groups of individuals who either gain or lose connectivity or between modality edges. After adjustment for multiple hypothesis testing, we found no significant differences between the groups (Supplementary Table 5). A more detailed description of methods and results can be found in the Supplemental Methods.

### Inter- and intra-individual heterogeneity in edge structure during stress

**Fig. 3:**
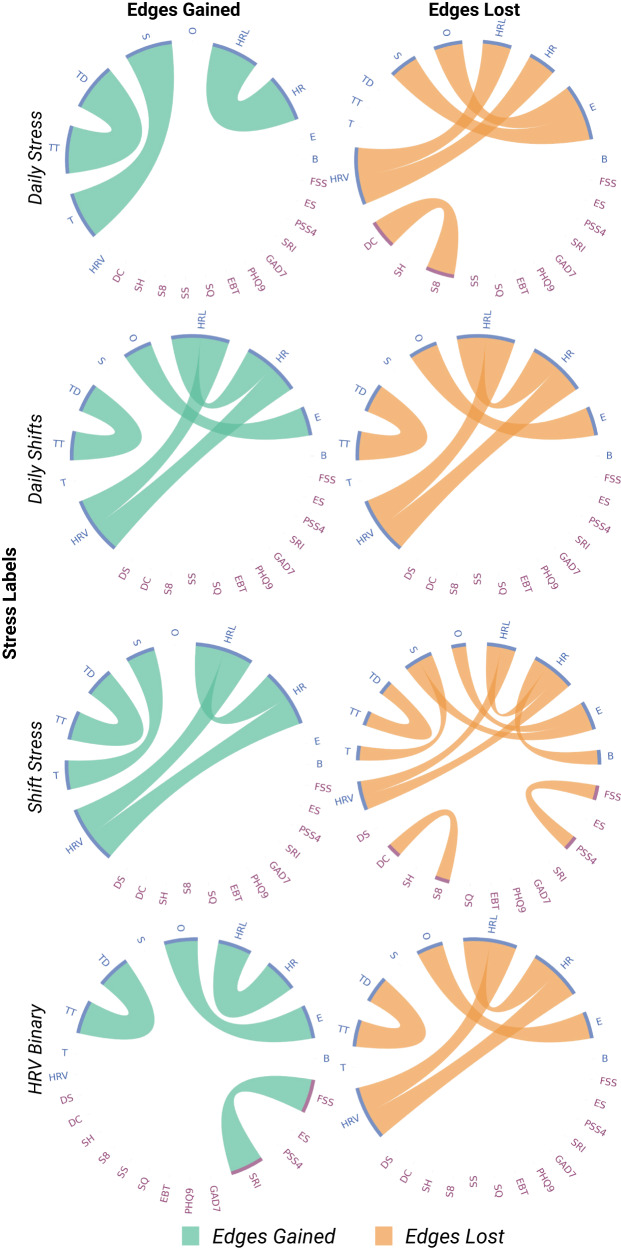
Chord diagrams representing edges frequently gained and lost at the population level across four different labels. Nodes (features) are on the outer ring, and colored chords represent edges that are frequently gained (green) and lost (orange) across the population, chord thickness is proportional to the frequency of the population where that respective edge is gained or lost. Nodes are colored according to the modality they are derived from (blue: Oura Ring, purple: Survey). Abbreviations for each feature/node can be found in Supplementary Table 1, along with a complete description of each feature.

We also looked at the specific edges between features that were gained or lost during stress. Figure 3 shows the population-level frequency of edges gained and lost during stress across all stress labels. Across all survey-derived stress labels (Daily Stressed, Shift Stress, Daily Shifts), edges between survey-derived features were more likely to be gained during stress than lost. For Daily Stressed and Shift Stress in particular, there were no survey-survey edges lost during stress. With the exception of HRV Binary, edges between HRV and HR (lowest or average) were more likely to be lost during stress than non-stress. However, it is important to note that though the changed edges in Fig. 3 were the most commonly observed in the population, there were no edges that were consistently gained or lost in all individuals (or even at 10% of individuals)—this suggests a high degree of inter-individual variability in edge changes.

## Discussion

Using causal discovery, we uncovered representations of stress as defined by the relatedness between features in causal graphs. Over 80% of our cohort showed significant changes in their graph structure on and off stress. This indicates a possible new method for evaluating states of stress in individuals by leveraging data from DHTs while still accounting for heterogeneity within and between individuals.

Our findings indicate that combining multiple stress definitions can lead to a more comprehensive understanding of stress. While prior works have explored stress using DHT data^12^, they rely on single “gold-standard” labels indicating when people are stressed. Such labels are typically derived from subjective self-reported measures. Unfortunately, other recent works have shown that such one-size-fits-all approaches to stress indication are insufficient^8^. Swain et al. note the clear differences between underlying latent mental states and actual observable measures, indicating the need for broader measures of complex states like stress. We therefore deliberately considered multiple definitions of stress, measured using different modalities. Our experiments then bolster the need for such heterogeneous stress definitions, as each definition we consider substantially differs from the others. Not only do we provide significant external validation of this prior work using innovative methods, we also describe the time-dependent correlation (or lack thereof) across these measures and how this varies between individuals. This characterization of the inter-individual heterogeneity of multiple time-varying labels indicates that future studies of stress will benefit from expanding their definitions of stress.

A core component of our work is embracing the individuality of stress; no two people experience stress exactly the same way. As we work with mental health constructs representing stress, prior work has shown that these measures can be highly heterogeneous between individuals^13,14^. However, most prior studies in digital health rely on population-level models, often training machine learning models to predict stress^10,15,16^. While such predictive models are surely useful in many cases, we pose that personalized understanding of stress is a key step toward better supporting individuals with stress and in better understanding stress in general. Though work in personalized modeling is not new, our results showcase a robust explanation for why population-level models fail (Supplementary Fig. 5). By learning robust individual causal graphs that represent the observed data, we are reconstructing the underlying data-generating process. Showcasing the inter- and intra-individual heterogeneity of these data-generating processes indicates that population-level models, which typically assume a single data-generating process, may fail.

Our results demonstrate the multimodal nature of stress; signals of stress derived from multiple sensors. In wearable data, this indicates some physiological coupling between signals (e.g., HR and HRV are expected to have an inverse relationship)^17^. In survey data, this may indicate consistency bias in self-reported measures along with associations between psychological factors^18^. For example, a negative answer to a survey question may propagate to their other answers and some measures are expected to be highly correlated, like depression and anxiety. In addition, the lack of connections between wearable and survey data indicates poor concordance between physiological and subjective measures. This suggests that stress requires multimodal assessments to capture its breadth. In particular, it is likely that the Oura Ring and Surveys are measuring different aspects of stress, with some overlap within the sensor type. Lastly, we were unable to identify clinically relevant insights explaining the graph changes occurring on and off stress. This may be the result of individuals not being inherently clusterable and rather existing on a continuous spectrum of stress representations or perhaps the variables explaining graph changes are simply not collected in our dataset. We leave this as an extension for future researchers.

Our dataset is unique and rich; however, it does present some limitations that are worth considering. Our data represent a unique population: frontline healthcare workers during the COVID-19 pandemic. As a result, our findings may not generalize to other populations. In addition, there are likely confounders. First, individual-level confounding may have a strong impact on the self-reported stress labels, and therefore the causal graphs we learned. Such confounding may occur due to past stress history, cultural-perceptions of stress, or the presence or absence of stress-reducing interventions. Second, the PC algorithm ignores unobserved confounding (i.e., features that are relevant in the causal structure but not measured). Such confounding is a major challenge in any causal discovery setting or observational study as it is infeasible to capture all relevant features.

Limitations also arose from the sensors used in the study. Our Oura Ring data were collected at night. This was because the Oura Ring in 2020 only captured nightly measures, and daytime sensors were not worn during shifts due to infection control reasons. Nighttime data may limit access to stress signals found in daytime data. However, having many nights of data over many individuals may efficiently control for the confounders present during the day. Further, Oura Ring data may also not be a complete set of physiologically relevant variables for stress. For example, electrodermal activity (EDA) is a widely recognized measure of acute stress, but is not captured by the Oura Ring. Another limitation is the different sampling frequencies of our different features. The surveys were designed to limit survey burden each day, so many surveys were only administered weekly, biweekly, or monthly. Since the sensor data was at the daily level, we imputed the lower-frequency measures. Such imputation may introduce bias into our results, though we describe ways that we worked to mitigate these biases in our results. As we ensured that missingness rates were no different during and off stress in the vast majority of individuals, we do not believe that missingness is a major driver of these results. Regardless of the difficulties in modeling multimodal data collected at varying frequencies, we believe that the diversity of measures we used in our study served to better capture latent representations of stress. Having a set of poorly correlated modalities reinforces the need for multimodal stress definitions spanning both subjective and objective measures.

In conclusion, our work provides valuable insights into how stress is an altered state using multimodal DHT data. This is a major step in understanding the complexity of stress representation in wearable data. For future work in this area, we showcase the difficulty in quantifying stress, owing to individual-level heterogeneity as well as poor ground-truth labels. To summarize, multiple stress labels are essential in capturing the heterogeneity of stress; multiple modalities (wearables, surveys, etc.) are required to learn robust representations of stress; and individualized modeling is required to capture the vast inter- and intra-individual heterogeneity in stress. As wearable devices become a part of routine clinical care, our robust characterization of inter- and intra-individual heterogeneity suggests that the metrics that clinicians might use to assess and treat stress should reflect this heterogeneity.

## Methods

### Study design

We used data from the Stress and Recovery in Frontline COVID-19 Healthcare Workers Study (Clinical Trial Registration: NCT04713111)^4^. The study followed 365 nurses for 4–6 months between March and December 2020, using active data from a smartphone app and passive data from an Oura Ring. Individuals also annotated when they worked, which we expected to coincide with periods of high stress. In addition, this study took place during the early stages of the COVID-19 pandemic, which likely heightened workplace stress. Individuals provided written, informed consent via the study app.

### Study measures and data preprocessing

Our data included physiological measures such as heart rate (HR), heart rate variability (HRV), relative body temperature, and sleep quality metrics from an Oura Ring worn nightly by individuals. Self-reported surveys and active task measures were collected daily, weekly, biweekly, and monthly from a smartphone app. These active tasks measured various components of stress, mental health, physical health and each individual’s psychosocial context. Together, these data represent a multivariate, multimodal time series for each individual. A full list of measures and their descriptions can be found in Supplementary Table 1. A more detailed dataset description including specific survey questions can be found at https://www.synapse.org/#!Synapse:syn24994804/wiki/.

To characterize periods of stress, we need labels from the data to indicate when a participant is stressed. However, stress can be subjective, so relying solely on participant’s self-reported periods of stress is prone to biases^8^. Therefore, we considered multiple definitions, aiming to capture a broader picture of stress with four measures: “Daily Stress” (a self-reported daily binary indicator of stress), “Daily Shifts” (a self-reported daily binary indicator of clinical shifts worked), “Shift Stress” (a self-reported daily binary indicator of stressful shifts worked), and “HRV Binary” (where stress is defined as the bottom quartile of an individual’s HRV distribution, and non-stress is defined as the top quartile). We then constructed a binary label (“Stress” vs. “Non-Stress”) according to the four definitions above at each day in each participant’s time series. By using multiple labels that span both subjective and objective measures of stress, our work takes a big step toward understanding the complex and latent nature of stress.

### Analysis

#### Data preprocessing

We *z*-score normalized each individual’s time series data. When data is missing for up to a week, we linearly interpolate missing values, and fill remaining missing values with the individual’s mean. No imputation was done for the stress labels.

#### Data exploration

Due to the variable measure frequencies across the surveys, survey completion rates were calculated based on their expected frequency (i.e., a monthly administered survey should only have values once a month for each individual). We also looked at missingness rates for the Oura Ring by determining the number of days with values divided by the total number of expected days of observation for each individual. Missingness and survey incompleteness could be due to a variety of reasons: not charging/wearing the smart ring, failing to complete a set of surveys, or sensor issues.

In order to better inform the causal discovery approach we took, we also examined the periodicity found within our time series data. We used a Fast Fourier decomposition of each individual’s physiological signals (e.g., HR, HRV, Temperature, Respiratory Rate) to reveal the dominant periods. A distribution of dominant periods stratified by individual sex was generated across the measures (Supplementary Fig. 4). This allowed us to identify weekly and monthly cycles that our data showed, as well as to characterize the inter-individual heterogeneity in these cycles, specifically how sex impacts these results.

#### Heterogeneity of stress labels

In order to demonstrate the importance of multiple stress labels, we computed the overall agreement between each pair of stress labels using the Jaccard similarity score for each individual. The Jaccard score ranges from 0 (complete misalignment) to 1 (perfect alignment). For example, if the Jaccard score across the two labels Daily Shifts and Daily Stressed is 1, then the times representing stress are the same across both measures.

#### Robust causal discovery

We used graph-based causal discovery in order to construct representations of potential stress states using multivariate data. These methods are inherently interpretable and allow us to model the data in a simple, graph-based representation. The graphs represented the relatedness of features across different stress states, which allowed us to help answer our scientific objectives—which features are highly related during stress compared to non-stress, and how this differs within and between individuals?

We used the PC algorithm, a well-established method for causal discovery (Supplementary Note 1)^9^. The algorithm uses independent and identically distributed (iid) sampled observations to generate a Directed Acyclic Graph (DAG), which represents the causal Bayesian network explaining the observations. Each node of the DAG represents a feature, while the edges are causal relations. For example, an edge from HR to HRV suggests that HR causes changes in HRV. However, the traditional PC algorithm does not handle time series. To satisfy the iid-requirement of the algorithm, we sampled discontinuous windows (Supplementary Note 2). We also only used undirected graphs learned from the algorithm, to focus on feature relationships as opposed to causal directions, and as edge orientation can be inconsistent in low sample sizes. This approach was done for each individual on and off each stress label, yielding an undirected graph describing the relationships between all features for each individual for each stress state. This approach is bolstered by the fact that recent work shows that using PC on samples from a time series can consistently identify the underlying causal graph of multivariate time series, when the time series is stationary and has strong mixing properties^19^. Our analysis of the data reveals this stationarity over several months of observation, and we assume the underlying data-generating process has these strong mixing properties.

The graphs obtained using PC could be highly influenced by sample size, samples used to construct the graph, and on their own lacked the ability to represent confidence via statistical hypothesis testing. Therefore, we employed several techniques to ensure the robustness of these graphs to these factors. To control for sample size, we limited the number of samples used to generate graphs on and off stress. We also used bootstrapping, uniformly sampling time series windows with replacement 100 times, resulting in 100 pairs of stress and non-stress graphs per participant. Lastly, to ensure that resultant differences between graphs on and off stress were statistically significant, we introduced a reference distribution to compare against. We repeated the 100 iterations of bootstrap sampling without considering stress labels. This creates two distributions of graph pairs, one sampled according to a stress label (Stress/Non-stress) and one sampled at random (reference), for each individual. We then measured the similarity (Methods) between each pair of graphs in the stress/non-stress and the reference distributions.

**Fig. 4:**
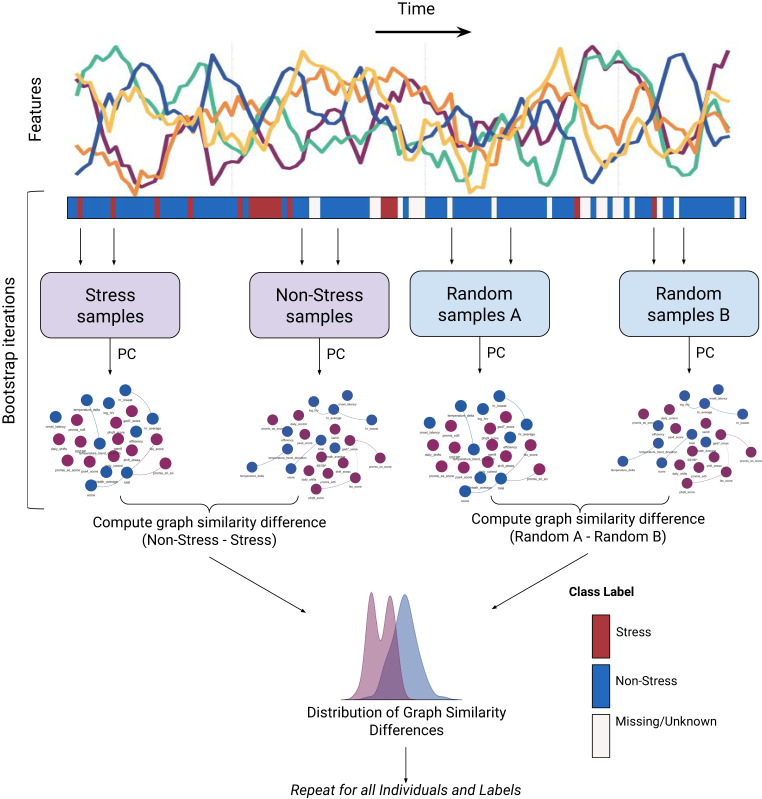
Visual description of sampling method and generation of reference distributions for connectivity differences. Multivariate time series features (multicolors: temperature, HR, HRV, etc…) change over time and a binary stress label is shown underneath (red: stress, blue: non-stress). Bootstrap sampling is used to generate iid observations from the time series data, samples are taken from Stress and Non-stress as well as at random (agnostic to stress label), in order to compare stress to a random sampling. Causal graphs representing the causal structure underlying the observations (blue circles: Oura Ring features, purple circles: Survey features) are then generated across the 100 bootstrap iterations, which are then compared using a graph similarity measure. Graph similarity refers to a series of metrics we used to compare graphs during Stress and Non-stress (see Methods).

Finally, to identify significant differences on and off stress, we employed a two-sample Kolmogorov–Smirnov (KS) test. We identified individuals whose distributions of graph similarities for stress/non-stress distribution are different from the reference distribution. We adjusted the *p* values across individuals for multiple hypothesis testing using the Benjamini–Hochberg procedure to control the false discovery rate^20^. Overall, we generated one graph per individual on and off stress (across four stress labels) with confidence they were robust to sampling effects and the ability to infer the statistical significance of these graphs compared to random sampling. The complete analysis pipeline for a single individual is visualized in Fig. 4.

#### Graph similarity metrics

We used three graph similarity measures to compare stress and non-stress causal graphs:

- Connectivity: the logarithm of the average node connectivity of all nodes in a graph. This metric is highest when the graph is fully connected^21^.
- Intersection/Union: the number of edges that are identical in each graph divided by the total number of unique edges. This compares the specific edge changes between the two graphs even if the connectivity does not change.
- Between Modality Edges: the number of edges connecting different modalities (Oura Ring or Survey). This is another measure that describes the types of edges that differ between the graphs.

#### Ethics

The original Stress and Recovery study and this analysis were approved by the Institutional Review Board Advarra (4UCOVID1901, Pro00043205).

### Reporting summary

Further information on research design is available in the Nature Research Reporting Summary linked to this article.

### Supplementary information

Supplementary Material

Reporting Summary

## Data Availability

The data used in this study were collected by 4YouandMe, a non-profit that makes all study data available to qualified researchers. The Stress and Recovery dataset is currently available on the Synapse platform (synapse.org) at Sage Bionetworks (https://sagebionetworks.org) and can be freely accessed by any researcher who becomes “qualified” by becoming a registered and certified Synapse user (https://help.synapse.org/docs/User-Account-Tiers.2007072795.html) and by meeting the specific conditions of use that require submitting an intended data use statement alongside an IRB approved protocol.

## Code availability

Codes to reproduce results and experiments in this study are made available via GitHub (https://github.com/sujaynagaraj/stress_recovery_public).
