# Supplementary material for: Dissecting the heterogeneity of “in the wild” stress from multimodal sensor data
Source: NPJ Digit Med. 2023 Dec 20;6:237. doi: 10.1038/s41746-023-00975-9 (PMC10733336; doi:10.1038/s41746-023-00975-9)
Supplement: Supplementary file 1 — Supplementary Material [file 41746_2023_975_MOESM1_ESM.docx]

**Supplementary Material**

**Table of Contents**

1. **Supplementary Notes**
   1. **PC Algorithm**
   2. **Sampling Algorithm**
   3. **Graph Visualization**
2. **Supplementary Methods**
   1. **Sanity Checks**
   2. **Variability in Predictive Model Performance**
   3. **Clustering of Causal Changes**
3. **Supplementary Tables**
4. **Supplementary Figures**

**Supplementary Notes**

***Supplementary Note 1***

A graph G = (V, E) consists of a vertex set V = X1, ..., Xp and an edge set E. The vertices represent random variables and the edges represent relationships between pairs of variables. We use the notation Xi ⊥⊥ Xj |S to indicate that Xi is independent of Xj given S, where S is a set of variables not containing Xi and Xj . If S is the empty set, we simply write Xi ⊥⊥ Xj . If Xi ⊥⊥ Xj |S, we refer to S as a separating set for (Xi, Xj ).

The PC algorithm is a constraint-based causal analysis framework which takes into samples of random variables, assuming no time series order and returns a Directed Acyclic Graph (DAG). The algorithm requires two crucial steps: 1) a skeleton learning step, where the output is an undirected graph, and 2) orienting the edges to form an equivalence class of DAGs. Starting with a complete undirected graph, a skeleton is rendered after pairwise conditional independence testing of every vertex - where an edge between Xi and Xj is deleted if Xi ⊥⊥ Xj |S. In the first iteration, S is the empty-set, sowe only test for marginal independence between nodes. After this iteration, different sizes of S are tested. For our experiments, we set the maximum size of S to two (ie:Xi ⊥⊥ Xj |Xk, Xl). For each conditional independence that is found, the corresponding edge between Xi and Xj is removed, and its conditioning set S is saved as a separating set (Xi, Xj ) and separating set (Xj , Xi). Once the skeleton is learned, the algorithm orients colliders to generate a DAG. This process involves a set of specific rules for each pair and triplet of adjacent vertices. The PC algorithm is sound and complete, however the order of vertices that are tested for conditional independence can have a major impact on the final graph. As such, a newer version of the algorithm called “PC Stable” was developed to address this issue, and it produces a consistent graph upon each iteration. We used the Python

Pgmpy implementation of this algorithm. For our experiments and visualizations, we ignore the direction of specific edges, due to the relatively small size of our dataset, and instead we generate an undirected graph, where edges correspond to the marginal dependence between nodes. The selection of a conditional independence test for edge deletion is crucial. For continuous variable data, a conditional independence test derived from Pearson partial correlation can be used. When S is the empty-set, simple Pearson correlation can be used. Where each test yields a correlation value and a p value, indicating the probability of an uncorrelated system producing a Pearson correlation at least as extreme as the one calculating. The partial correlation between Xi and Xj given a separation set S, is the correlation between the residuals eXi and eXj resulting from the linear regression of Xi with S and of Xj with S, respectively. As previously described, a hypothesis test to determine the statistical significance of this value can be used. As such, we can now use these measures for graph skeleton construction in Step 1 of the PC algorithm, where edges are removed if Xi ⊥⊥ Xj |S and p < 0.05. For our experiments, we ensured that at least 20 samples were used to generate each graph (Stress and Non-Stress). As a result, some individuals with few labels of stress were not included in the experiments.

***Supplementary Note 2***

1. First we define a binary stress label (ie: on shift would be Stress, and off shift would be Non-Stress). Each day of data is then labeled as one of three classes: Stress, Non-Stress, or NAN (to account for missing labels)
2. For each individual:
   1. For i in range(100) bootstrap iterations (stress-based sampling)
      1. For each timestep (day) iid sampling:
         1. For Non-Stress (label = 0) and Stress (label = 1):
            1. If the label for that day is a local singleton (ie: surrounded by days of different labels)

Keep that day’s observation for analysis

- - - - 1. If a sequence of two or three continuous days have the same label

Use the mean across all continuous days for analysis as one sample

- - - - 1. If a sequence of 4 or more days have the same label

Randomly sample half of days for analysis

- - 1. Compute two causal graphs using samples from Stress and Non-Stress, respectively
    2. Compute graph similarity measure comparing the two graphs
  1. For i in range(100) bootstrap iterations (random sampling)
     1. Randomly sample two sets of days without replacement (Random A and Random B)
     2. Compute two causal graphs using samples from Random A and Random B, respectively
     3. Compute graph similarity measure comparing the two graphs
  2. Compare the distributions of graph similarity measures across both sets of bootstrap iterations (stress-based sampling and random sampling) using a KS test

1. Multiple hypothesis testing

***Supplementary Note 3***

Using the bootstrap sampling method we described earlier for each individual and each stress label, we have 100 graphs for each of Stress and Non-Stress. Due to variance that will occur as a result of sampling, each of these graphs may have a slightly different set of edges. In order to estimate the most representative graph for each of Stress and Non-Stress, we construct a weighted, undirected graph, where the weight is the frequency the edge occurred in the 100 bootstrap iterations.

In order to visualize the edges gained and lost on each stress label, we generate a symmetric adjacency matrix using the weighted graphs on Stress and Non-Stress. We drop any edge that occurred less than 75% of the time in an individual, to get the most consistent edges. Then we compute the difference adjacency matrix (Non-Stress - Stress), to identify the change in edge frequency during Stress; edge weights (adjacency matrix values) that are negative indicate edges that are lost on Stress, and edge weights that are positive indicate edges that are gained on Stress. Then we count the number of individuals who had that edge. We generate an undirected weighted graph where the edge weights are the proportion of individuals who had that edge. For a cleaner visual, and to exclude outliers, we drop edges that change in less than 5% of the population.

**Supplementary Methods**

*Sanity Checks*

As an initial pass to evaluate the validity of our dataset, we identified several sanity checks to recreate previously reported or expected findings. One such expected relationship in the data is that of heart rate and heart rate variability, which studies have shown tend to possess an inverse relationship and that is also corroborated here. Another sanity check was the relationship between Oura Ring physiologic and stress labels (Supplemental Figure 1). We found inter-individual variability in how distributions of Oura Ring measure values change conditioned on different stress labels (Supplemental Figure 2). For many individuals, there were notable differences in Oura Ring measure distributions on vs off stress. Our causal analysis described below further unpacked the complexity and multivariate nature of these relationships.

*Variability in Predictive Model Performance*

As a motivating example, we construct simple classifiers using XGBoost (default parameters) to try to use the Oura Ring and Survey features to predict three of our binary stress labels (Daily Stress, Daily Shifts, Shift Stress). We do not use HRV Binary as a task, as it is highly correlated with the Oura Ring features and thus achieves near perfect performance. We use the same preprocessing pipeline for our causal discovery approach and we split the dataset 80:20 into a training and testing set. This split was done in order of time and for each participant (ie: the first 80% of data points from Participant A in time order go to the training set and the last 20% of data points from Participant A go into the testing set). This also helps to ensure that each participant is represented in the training and the testing sets. Once the model is trained, we evaluate its performance on the testing set while stratifying for each unique participant. This gives us a distribution of test set performance metrics across the population. Supplemental Figure 5 illustrates the wide heterogeneity in model performance across different individuals. The bimodal peaks, particularly for Daily Stress and Shift Stress, suggest that there are some individuals where we can predict quite well and some individuals where we cannot. This serves to echo the importance of individualized modelling. Our causal discovery analysis aims to unpack why this is the case.

*Clustering of Causal Changes*

Our use of graph connectivity measures described in Methods give us a nice way to cluster individuals based on how their causal structure changes. For example, with respect to the node connectivity and between modality edge measures, we can split individuals into those who have overall gains or losses in connectivity. Note that this analysis is not possible with the Intersection/Union measure as this measure is symmetric and does not have a notion of direction (ie: gain or loss).

With these natural groupings, we run statistical tests to determine if there are differences in overall enrichment between the groups with respect to various categorical and continuous time-invariant features we collected in our study. For categorical features, we use a Chi-square test to determine if the proportion of each time-invariant feature is different between two groups. For continuous features, we use a T-test assuming unequal variance. A full list of the measures is found in Supplemental Table 4. Note that we selected a set of features that covered a broad range of important categories (ie: mental and physical health, prior stress history, as well as protective and risk factors) that also had sufficient variability amongst individuals. For example, the presence of mental health disorders such as Psychosis, which was present in <5% of the population, was excluded.

First we report the statistically significant features in Supplemental Table 5. We can see that there are some differences between groups based on various interesting measures. However, once we apply post-hoc correction for multiple hypothesis testing, we find that these differences vanish, and we are only left with only two statistically significant findings: those who have an overall Gain in between modality edges for the Daily Shifts label tend to be older (p = 0.0046), and those who have a gain in node connectivity for the Shift Stress label tend have a greater Life Events score (p = 0.0068).

This lack of significantly significant features may be a result of the sheer number of hypotheses that we test and the ensuing effect of post-hoc correction. It may also be the result of these individuals not being inherently clusterable and rather existing on a continuous spectrum of stress representations or perhaps the explanatory variables are simply not collected in our dataset. We leave this as an extension for future researchers in our own dataset which we make available or outside of this setting. We believe the methods we have developed to learn these robust stress representations may lend themselves to future clustering attempts.

**3) Supplementary Tables**

***Dataset Features***

| **Feature Name** | **Figure Abbreviation** | **Source** | **Description (frequency)** |
| --- | --- | --- | --- |
| Breath Average | B | Oura Ring | Average respiratory rate (nightly) |
| Efficiency | E | Oura Ring | Total percentage of sleep period spent asleep (nightly) |
| HR Average | HR | Oura Ring | Average heart rate (nightly) |
| HR Lowest | HRL | Oura Ring | Lowest heart rate (nightly) |
| Sleep Onset | O | Oura Ring | Time to first 5 minutes of prolonged sleep (nightly) |
| Sleep Score | S | Oura Ring | Representation of overall sleep quality (nightly) |
| Temperature Change | TD | Oura Ring | Change in skin temperature from start of sleep to end of sleep (nightly) |
| Temperature Trend | TT | Oura Ring | Skin temperature deviation from long term average (nightly) |
| Total Sleep | T | Oura Ring | Total time spent asleep (nightly) |
| HRV | HRV | Oura Ring | Log-transformed average heart rate variability (nightly) |
| Stress | DS | Survey | Binary indicator of extreme stress (daily) |
| Control | DC | Survey | Percentage of feeling in control (daily) |
| Shifts | SH | Survey | Binary indicator of clinical shifts worked (daily) |
| SAM8 | S8 | Survey | Categorical stress level during shift (daily) |
| Shift Stress | SS | Survey | Binary indicator of high shift stress (daily) |
| Sleep Quality | SQ | Survey | Categorical subjective sleep quality assessment (weekly) |
| EBT | EBT | Survey | Continuous measure of emotional bias (every other day) |
| PHQ9 | PHQ9 | Survey | Clinically validated measure of depression (biweekly) |
| GAD7 | GAD7 | Survey | Clinically validated measure of anxiety (biweekly) |
| Impaired Sleep | SRI | Survey | Subjective assessment of sleep related impairment (weekly |
| PSS4 | PSS4 | Survey | Subjective assessment of perceived stress (weekly) |
| Emotional Support | ES | Survey | Subjective assessment of available emotional support (weekly) |
| FSS | FSS | Survey | Subjective assessment of fatigue severity (weekly) |

**Supplemental Table 1.** List of available measures used for analysis. Includes measure name, acronym used in figures, modality-type, and description of the measure along with sampling frequency.

| **Characteristic** | **Mean/Frequency (n)** |
| --- | --- |
| Average Age (years) | 36 +/- 10 (n=365) |
| Average Weight (lbs) | 167 +/- 40 (n=353) |
| % Female | 89% (n=365) |
| % Caucasian | 83% (n=365) |
| % Any Chronic Health Condition* | 57% (n=353) |
| % Any Mental Health Condition** | 59% (n=353) |
| Average Adverse Childhood Experience (ACE) Score*** | 1.8 +/- 2.2 (n=346) |
| Average Post Traumatic Stress Disorder (PTSD) Score*** | 31.8 +/- 11.0 (n=130) |
| Alcohol Use (at least 2-3 x per week) | 41% (n=350) |
| Smoking (at least 2-3 x per week) | 3% (n=350) |
| Exercise (at least 2-3 x per week) | 50% (n=350) |

**Supplemental Table 2**. Summary statistics describing cohort demographics where *n* is the total number of individuals where data for that measure was available. +/- represent Standard Deviation *Having or have ever had any of: diabetes, obesity, heart disease, circulation or blood pressure problems, lung or breathing problems, arthritic or rheumatic problems, kidney disease, liver disease, allergies, chronic pain, digestive system problems, ENT problems, neurological problems, or other (self-reported). ** Having a diagnosis of any of: mood disorder, anxiety disorder, psychotic disorder, eating disorder, neurodevelopmental disorder, sleep disorder, suicidal ideation or attempt, learning disability, or other (self-reported). *** ACE and PTSD scores were calculated based on validated surveys designed to measure these constructs (ACE quiz and PCL-10, respectively).

| **Survey Measure** | **Frequency** | **% Completed** |
| --- | --- | --- |
| Daily Stress | Daily | 70% +/- 20% |
| SAM | Daily | 46% +/- 14% |
| PSS4 | Weekly | 79% +/- 18% |
| PROMIS - SD | Weekly | 79% +/- 18% |
| PROMIS - SRI | Weekly | 75% +/- 19% |
| FSS-9 | Weekly | 77% +/- 19% |
| PHQ-9 | Biweekly | 85% +/- 16% |
| GAD-7 | Biweekly | 85% +/- 16% |
| PROMIS ES | Monthly | 95% +/- 11% |

**Supplemental Table 3:** Completeness statistics for survey observations. % Completeness represents the total number of observations divided by the expected number of observations during the study duration at the individual-level (ie: a monthly survey over 6 months should have 6 observations), where average and +/- standard deviation is computed across different individuals.

| **Feature Name** | **Description** | **Feature Type** |
| --- | --- | --- |
| Age | Self-reported Age of participant at start of study | Continuous |
| Weight | Self-reported Weight of participant at start of study | Continuous |
| ACE Score | Score calculated from self-reported Adverse Childhood Experiences (ACE) Survey. ACE survey includes Yes/No responses to questions regarding early adverse life events that may have occurred in the first 18 years of life. | Continuous |
| PTSD Score | Score calculated from self-reported Trauma - PTSD Survey. This includes a likert scale of responses to a list of problems and complaints that people sometimes have in response to stressful life experiences that best indicate how much they are bothered by those problems over the past month. | Continuous |
| Life Events Score | Score calculated from self-reported Life Events Checklist 10-Item Survey. The Life Events Checklist 10-Item survey includes multiple choice and Yes/No questions regarding events that commonly happen in people’s lives. | Continuous |
| Gender | Self-reported Gender of participant. | Categorical |
| Past Medical History | Having or have ever had any of: diabetes, obesity, heart disease, circulation or blood pressure problems, lung or breathing problems, arthritic or rheumatic problems, kidney disease, liver disease, allergies, chronic pain, digestive system problems, ENT problems, neurological problems, or other (self-reported). | Categorical |
| Mood Disorders | Having a diagnosis of any Mood disorder (e.g. Depression, Dysthymia, Bipolar disorder) | Categorical |
| Anxiety Disorders | Having a diagnosis of any Anxiety disorder (e.g. PTSD, OCD, Panic Disorder, Simple Phobia, Social Phobia, GAD, Agoraphobia) | Categorical |
| Any Mental Health Disorder | Having a diagnosis of any of: mood disorder, anxiety disorder, psychotic disorder, eating disorder, neurodevelopmental disorder, sleep disorder, suicidal ideation or attempt, learning disability, or other (self-reported) | Categorical |
| Alcohol Consumption | Self-reported alcohol consumption in the past month | Categorical |
| Smoking | Self-reported tobacco smoking or vaping in the past month. | Categorical |
| Exercise | Self-reported exercise in the past month. | Categorical |

Supplemental Table 4: Description of time-invariant features used for comparisons in cluster enrichment as well as if they were Categorical or Continuous. For a full description of the specific survey questions asked to elicit each feature, please see the link to the Dataset in Methods.

|  | **Graph Similarity Measure** | |
| --- | --- | --- |
| **Stress Label** | **Connectivity** | **Between Modality Edges** |
| *Daily Stressed* | None | *↑* Weight  *↑* ACE Score |
| *Daily Shifts* | *↑* ACE Score | *↑* Age  *↑* Any Mental Health Disorder |
| *Shift Stress* | *↑* ACE Score  *↑* PTSD Score  *↑* Life Events Score | None |
| *HRV Binary* | ↓ Life Events Score | None |

Supplemental Table 5: For each Stress Label and Graph Similarity Measure, table shows if there was a statistically significant difference in time-invariant features for individuals who have a Gain in that Similarity Measure On Stress. This analysis was only calculated on the population that had a statistically significant change in graph similarity on/off stress compared to their reference distribution (see Table 1) and does not include post-hoc multiple hypothesis testing correction. For example, individuals who had an overall Gain in graph connectivity during Daily Shifts had a higher average ACE score.

**4) Supplementary Figures**


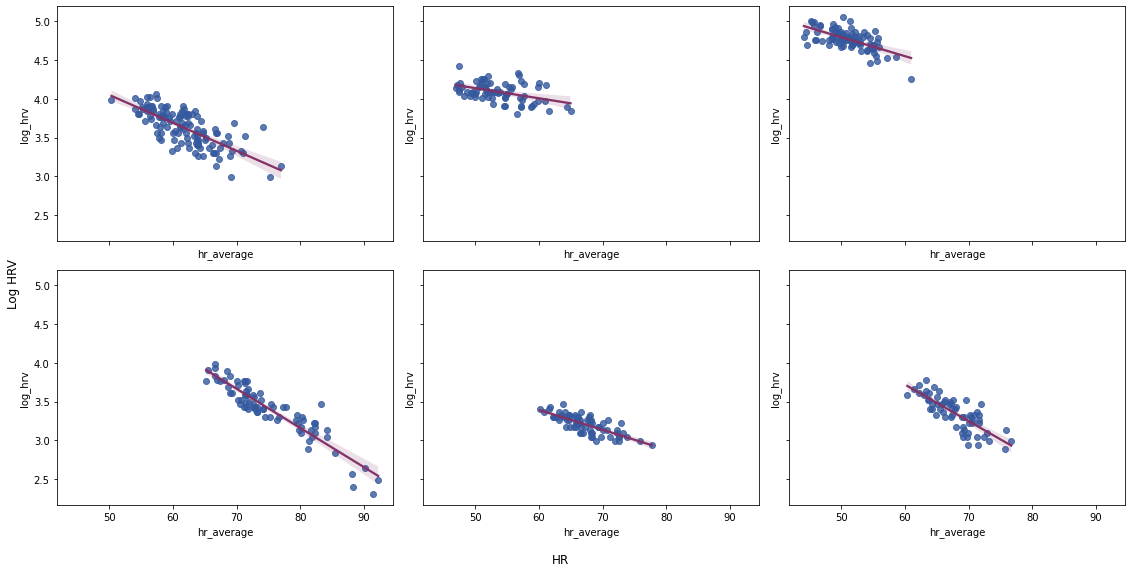


Supplemental Figure 1. Inverse relationships between HRV and HR across six random individuals.


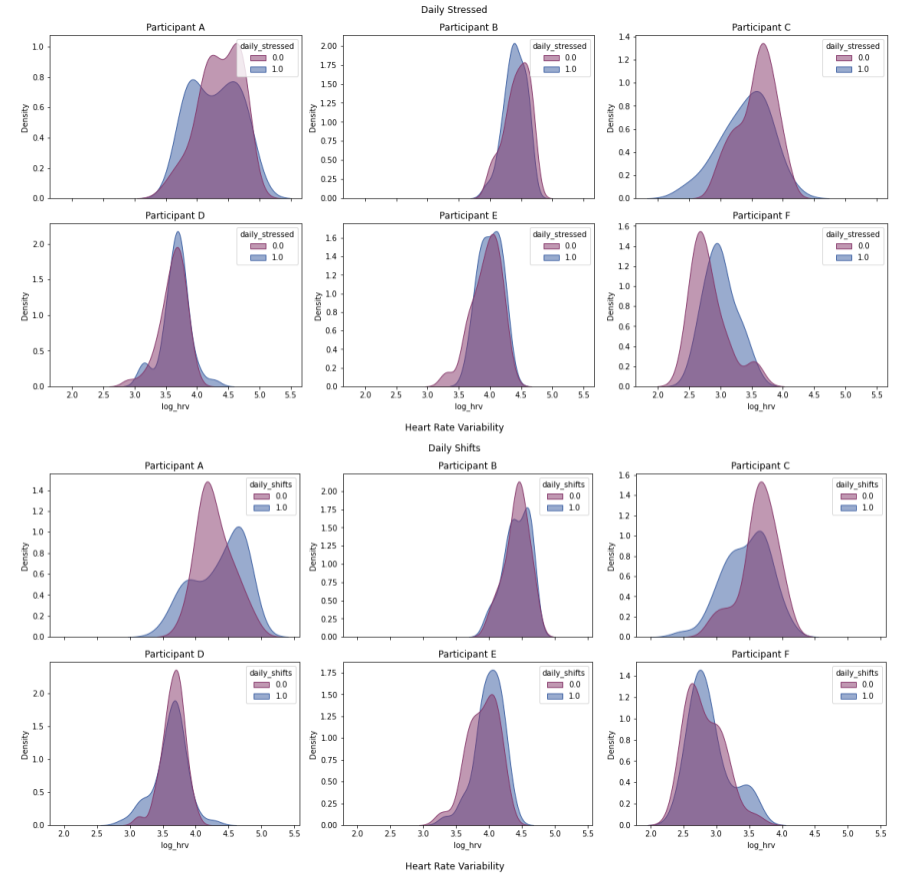


Supplemental Figure 2. Probability density plots showing distribution differences of Heart Rate Variability on (1.0) and off (0.0) Daily Stressed (A) & Daily Shifts (B) for six random individuals. Figure highlights the inter and intra-individual heterogeneity in feature distributions conditioned on stress.

~~
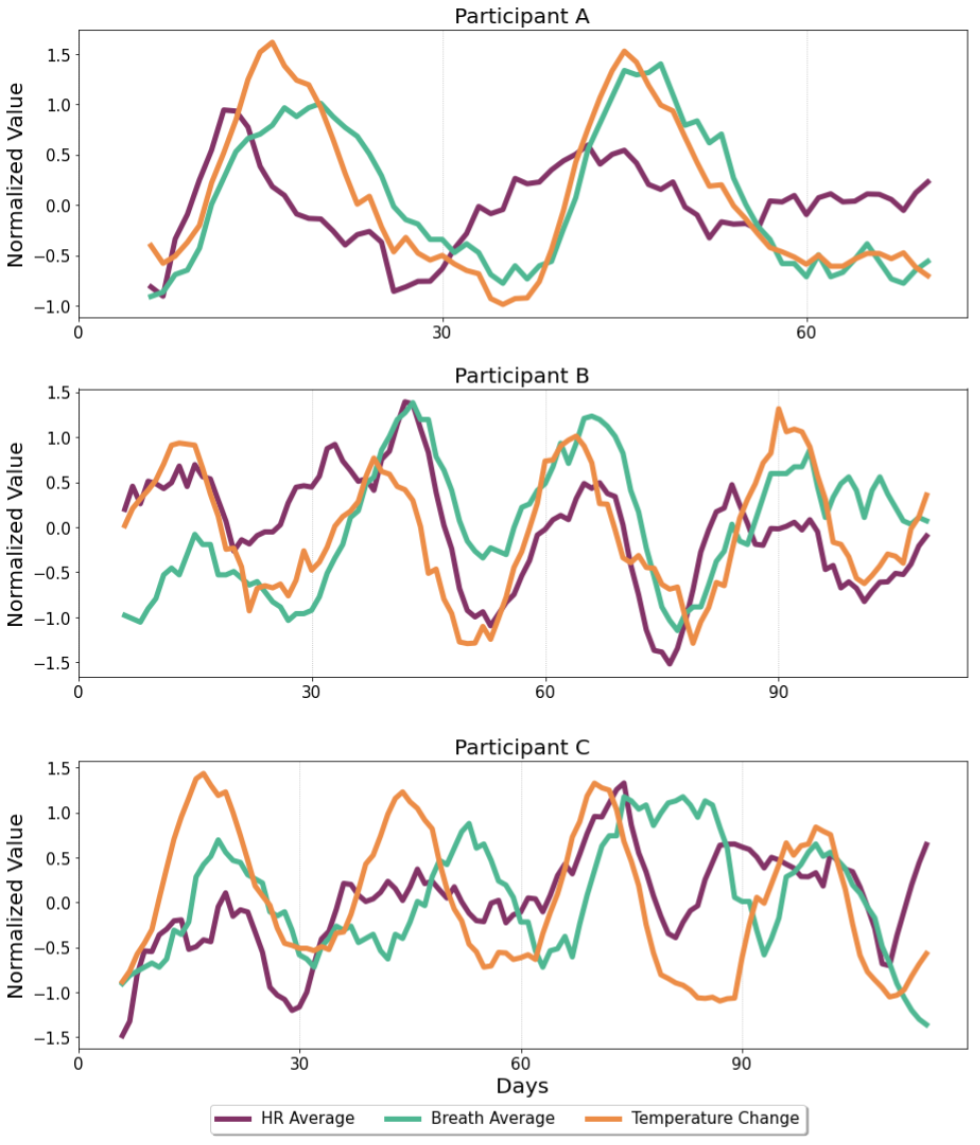
~~

Supplemental Figure 3. Normalized rolling averages (7 day window) for HR Average, Breath Average, and Temperature over study duration for three random participants. Figure highlights the strong periodicity seen in our data, with particularly strong periods every 30 days.


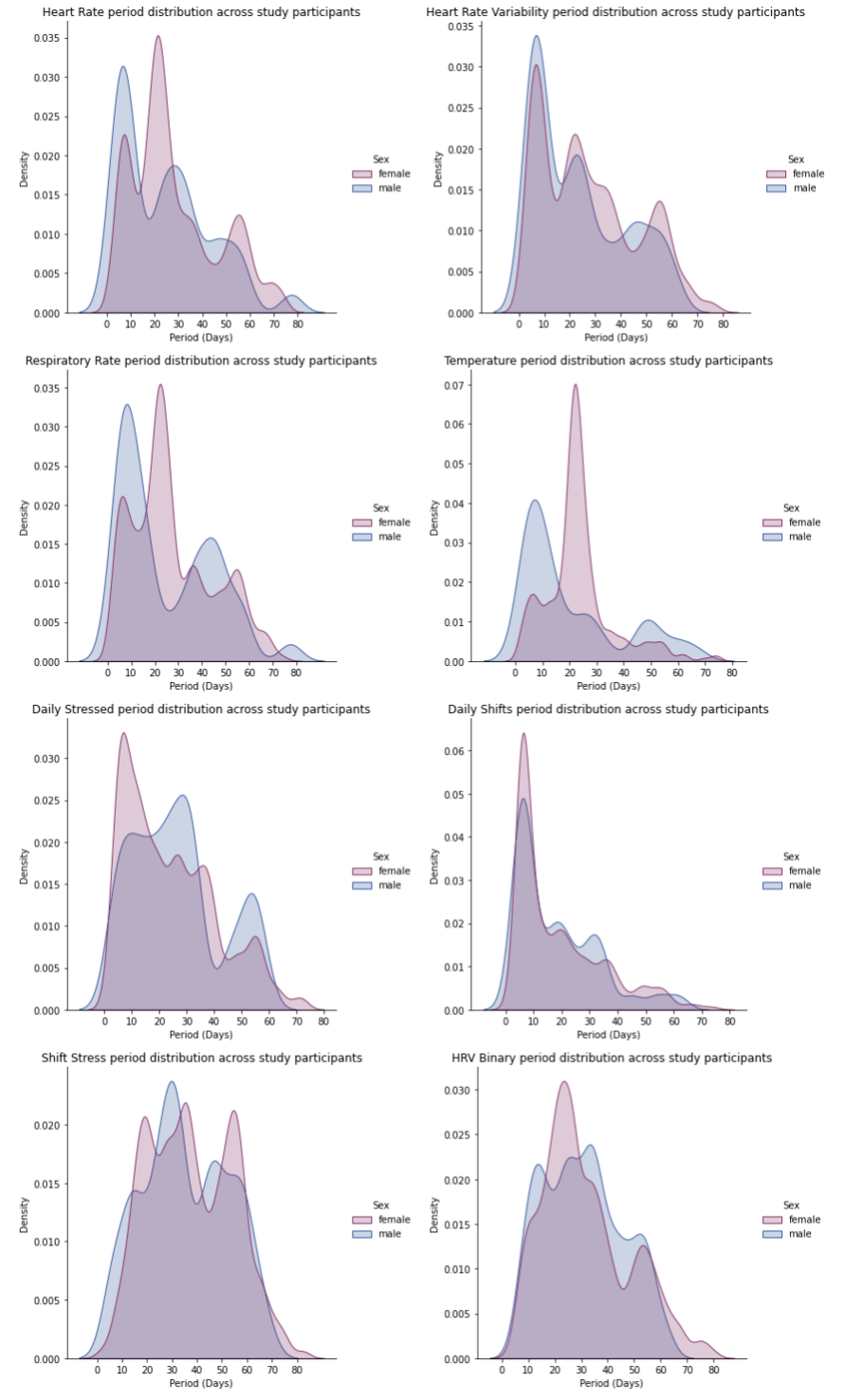


Supplemental Figure 4. Distribution of peak periods after FFT analysis across four Oura Ring features (HR, HRV, Temperature, and Breath Average) and all four stress labels, stratified by sex. Distributions are across all individuals. Figures show normalized probability density plots, high density around a specific period implies increased frequency of individuals having that peak period.


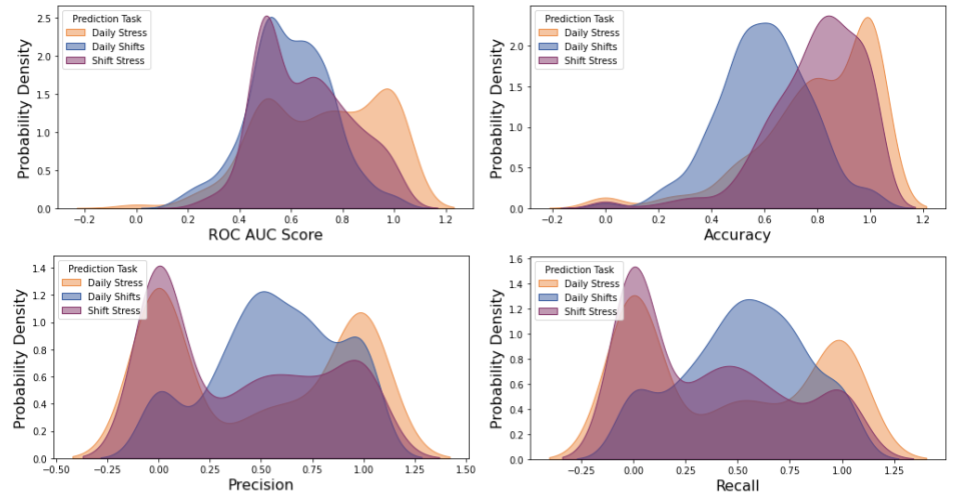


Supplemental Figure 5: Testing set performance distribution across unique individuals of XGBoost Classifier trained on the entire population for three different tasks (colored hues). Kernel density estimate plots are shown which represents a valid probability distribution, and why the x-axis can be negative at times. We use this to illustrate the heterogeneity in model performance across the population.
