# Supplementary material for: Dissecting the heterogeneity of “in the wild” stress from multimodal sensor data
Source: NPJ Digit Med. 2023 Dec 20;6:237. doi: 10.1038/s41746-023-00975-9 (PMC10733336; doi:10.1038/s41746-023-00975-9)
Supplement: Supplementary file 2 — Reporting Summary [file 41746_2023_975_MOESM2_ESM.pdf]

nature portfolio

Corresponding author(s): Sujay Nagaraj

Last updated by author(s): Sep 17, 2023

Reporting Summary

Nature Portfolio wishes to improve the reproducibility of the work that we publish. This form provides structure for consistency and transparency in reporting. For further information on Nature Portfolio policies, see our Editorial Policies and the Editorial Policy Checklist.

Statistics

For all statistical analyses, confirm that the following items are present in the figure legend, table legend, main text, or Methods section.

n/a	Confirmed	
☐	☒	The exact sample size (*n*) for each experimental group/condition, given as a discrete number and unit of measurement
☐	☒	A statement on whether measurements were taken from distinct samples or whether the same sample was measured repeatedly
☐	☒	The statistical test(s) used AND whether they are one- or two-sided<br>*Only common tests should be described solely by name; describe more complex techniques in the Methods section.*
☐	☒	A description of all covariates tested
☐	☒	A description of any assumptions or corrections, such as tests of normality and adjustment for multiple comparisons
☐	☒	A full description of the statistical parameters including central tendency (e.g. means) or other basic estimates (e.g. regression coefficient) AND variation (e.g. standard deviation) or associated estimates of uncertainty (e.g. confidence intervals)
☐	☒	For null hypothesis testing, the test statistic (e.g. *F*, *t*, *r*) with confidence intervals, effect sizes, degrees of freedom and *P* value noted<br>*Give P values as exact values whenever suitable.*
☒	☐	For Bayesian analysis, information on the choice of priors and Markov chain Monte Carlo settings
☐	☒	For hierarchical and complex designs, identification of the appropriate level for tests and full reporting of outcomes
☐	☒	Estimates of effect sizes (e.g. Cohen's *d*, Pearson's *r*), indicating how they were calculated

Our web collection on statistics for biologists contains articles on many of the points above.

Software and code

Policy information about availability of computer code

Data collection	Data is available publicly for those with IRB approval and we provide a link to the dataset, with instructions on how to access it.
Data analysis	We provide all the code required to reproduce the results and explore the data in a GitHub repository. We make use of existing software packages (details of all of which can be found in the GitHub repository)

For manuscripts utilizing custom algorithms or software that are central to the research but not yet described in published literature, software must be made available to editors and reviewers. We strongly encourage code deposition in a community repository (e.g. GitHub). See the Nature Portfolio guidelines for submitting code & software for further information.

Data

Policy information about availability of data

All manuscripts must include a data availability statement. This statement should provide the following information, where applicable:

- Accession codes, unique identifiers, or web links for publicly available datasets
- A description of any restrictions on data availability
- For clinical datasets or third party data, please ensure that the statement adheres to our policy

The data used in this study were collected by 4YouandMe, a non-profit which makes all study data available to qualified researchers. The Stress and Recovery dataset is currently available on the Synapse platform (synapse.org) at Sage Bionetworks (https://sagebionetworks.org) and can be freely accessed by any researcher who becomes ‘qualified’ by becoming a registered and certified Synapse user (https://help.synapse.org/docs/User-Account-Tiers.2007072795.html) and

by meeting the specific conditions of use that require submitting an intended data use statement alongside an IRB approved protocol.

Code to reproduce results and experiments in this study are made available via GitHub (https://github.com/sujaynagaraj/stress_recovery_public).

Research involving human participants, their data, or biological material

Policy information about studies with human participants or human data. See also policy information about sex, gender (identity/presentation), and sexual orientation and race, ethnicity and racism.

Reporting on sex and gender	The dataset we used collected self-reported Gender (with options being: Female, Male, Non-Binary/Third Gender, Other, and Prefer not to say). We describe the proportion of responses. As the population was mostly female, we do not do any analysis stratified by gender.
Reporting on race, ethnicity, or other socially relevant groupings	Participants self-reported race and ethnicity. We describe the proportions of responses but do not do any race or ethnically-stratified analysis as the population was quite homogeneous.
Population characteristics	Nothing to add here.
Recruitment	Frontline health care workers were recruited from May to August 2020. A multipronged recruitment approach was developed that involved engaging trusted leaders from organizations and sites with high outreach to our target population (eg, American Association of Critical Care Nurses), engaging supervisors at selected health care institutions, and a social media campaign (eg, Facebook, Twitter, and Linkedin). Tailored workplace-specific recruitment materials were developed and, during the enrollment period, assessed in real time how participants were learning about the existence of the study to understand the most effective recruitment strategies. Recruitment materials were distributed through workplace-specific newsletters and websites, and through our social media channels.
Ethics oversight	This study was approved by the Institutional Review Board, Advarra (4UCOVID1901, Pro00043205)

Note that full information on the approval of the study protocol must also be provided in the manuscript.

Field-specific reporting

Please select the one below that is the best fit for your research. If you are not sure, read the appropriate sections before making your selection.

☐ Life sciences ☒ Behavioural & social sciences ☐ Ecological, evolutionary & environmental sciences

For a reference copy of the document with all sections, see nature.com/documents/nr-reporting-summary-flat.pdf

Behavioural & social sciences study design

All studies must disclose on these points even when the disclosure is negative.

Study description	Quantitative, longitudinal cohort study
Research sample	Frontline health care workers were invited to participate including medical doctors, doctors of osteopathy, physician assistants, registered nurses, advanced practice registered nurses, and other allied health care workers. Inclusion criteria were must be working directly with patients with COVID-19, slated to do so within the next 2 weeks, or work routines have been moderately or extremely impacted by the COVID-19 pandemic; older than 18 years; able to speak, write, and read English; able to provide informed consent; no known SARS-CoV-2 current or past infection; and must own a personal iOS mobile phone (OS11 and above) with willingness to download and use the study apps and sync phone with all study sensors.
Sampling strategy	Frontline health care workers were recruited from May to August 2020. A multipronged recruitment approach was developed that involved engaging trusted leaders from organizations and sites with high outreach to our target population (eg, American Association of Critical Care Nurses), engaging supervisors at selected health care institutions, and a social media campaign (eg, Facebook, Twitter, and Linkedin). Tailored workplace-specific recruitment materials were developed and, during the enrollment period, assessed in real time how participants were learning about the existence of the study to understand the most effective recruitment strategies. Recruitment materials were distributed through workplace-specific newsletters and websites, and through our social media channels.
Data collection	Participants were mailed an Oura smart ring. The Oura Ring 2 is made of a light durable titanium shell and includes a temperature sensor, a gyroscope, a 3D accelerometer, and an infrared optical pulse sensor. There was a one-time setup process where the participant synced their ring to the Oura smartphone app that they were instructed to download. Throughout the study, the participant was instructed to open the Oura smartphone app to sync data off the ring over Bluetooth. The sensor collects a variety of nighttime data streams such as heart rate, heart rate variability, and objective sleep quality measures. Via the study app, participants were prompted to complete daily, weekly, every 2-week, monthly, and one time measures that involved sociodemographic factors, self-reported measures of daily perceived stress, intermediate signs of stress (sleep, mood, and cognition), additional health-related symptoms, influenza-like illness, individual characteristics, and cognitive active tasks
Timing	May 1, 2020, to November 20, 2020.

Data exclusions	Primary reasons for exclusion were being an Android user, prior COVID-19 infection, and no direct patient care
Non-participation	Exact statistics on follow up, retention, and drop out can be found in the original dataset description: https://formative.jmir.org/2021/12/e32165/
Randomization	No randomization was done. This was a cohort study.

Reporting for specific materials, systems and methods

We require information from authors about some types of materials, experimental systems and methods used in many studies. Here, indicate whether each material, system or method listed is relevant to your study. If you are not sure if a list item applies to your research, read the appropriate section before selecting a response.

Materials & experimental systems

n/a	Involved in the study	
☒	☐	Antibodies
☒	☐	Eukaryotic cell lines
☒	☐	Palaeontology and archaeology
☒	☐	Animals and other organisms
☐	☒	Clinical data
☒	☐	Dual use research of concern
☒	☐	Plants

Methods

n/a	Involved in the study	
☒	☐	ChIP-seq
☒	☐	Flow cytometry
☒	☐	MRI-based neuroimaging

Clinical data

Policy information about clinical studies

All manuscripts should comply with the ICMJE guidelines for publication of clinical research and a completed CONSORT checklist must be included with all submissions.

Clinical trial registration	NCT04713111
Study protocol	https://formative.jmir.org/2021/12/e32165/
Data collection	Frontline health care workers were recruited from May to August 2020. A multipronged recruitment approach was developed that involved engaging trusted leaders from organizations and sites with high outreach to our target population (eg, American Association of Critical Care Nurses), engaging supervisors at selected health care institutions, and a social media campaign (eg, Facebook, Twitter, and Linkedin). Tailored workplace-specific recruitment materials were developed and, during the enrollment period, assessed in real time how participants were learning about the existence of the study to understand the most effective recruitment strategies. Recruitment materials were distributed through workplace-specific newsletters and websites, and through our social media channels.
Outcomes	We performed secondary analysis on this dataset which was collected to assess the feasibility of a participant-centric digital approach to collect both participant-reported subjective and objective measured longitudinal high-resolution data on immediate stress responses, intermediate signs of stress, recovery from stress, and COVID-19 infectivity by engaging frontline health care workers in the use of digital sensors.
